# Dynamic Analysis of Gene Expression in Rice Superior and Inferior Grains by RNA-Seq

**DOI:** 10.1371/journal.pone.0137168

**Published:** 2015-09-10

**Authors:** Hongzheng Sun, Ting Peng, Yafan Zhao, Yanxiu Du, Jing Zhang, Junzhou Li, Zeyu Xin, Quanzhi Zhao

**Affiliations:** 1 Collaberative Innovation Center of Henan Grain Crops, Henan Agricultural University, Zhengzhou, China; 2 Rice Engineer Center, Henan Agricultural University, Zhengzhou, China; 3 Key Laboratory of Physiology, Ecology and Genetics Improvement of Food Crop in Henan Province, Henan Agricultural University, Zhengzhou, China; Wuhan University, CHINA

## Abstract

Poor grain filling of inferior grains located on lower secondary panicle branch causes great drop in rice yield and quality. Dynamic gene expression patterns between superior and inferior grains were examined from the view of the whole transcriptome by using RNA-Seq method. In total, 19,442 genes were detected during rice grain development. Genes involved in starch synthesis, grain storage and grain development were interrogated in particular in superior and inferior grains. Of the genes involved in sucrose to starch transformation process, most were expressed at lower level in inferior grains at early filling stage compared to that of superior grains. But at late filling stage, the expression of those genes was higher in inferior grains and lower in superior grains. The same trends were observed in the expression of grain storage protein genes. While, evidence that genes involved in cell cycle showed higher expression in inferior grains during whole period of grain filling indicated that cell proliferation was active till the late filling stage. In conclusion, delayed expression of most starch synthesis genes in inferior grains and low capacity of sink organ might be two important factors causing low filling rate of inferior grain at early filling stage, and shortage of carbohydrate supply was a limiting factor at late filling stage.

## Introduction

In rice seed development, the grain filling process is the most important factor related to the yield and quality of rice grains [1]. However, aside from genotype reasons, the grain filling rate varies according to the location on rice panicle. In rice panicle, earlier flowered spikelets on the upper apical primary rachis branches are called superior spikelets, and the later flowered spikelets on lower secondary rachis branches are called inferior spikelets [2–4]. The final grain weight and quality of superior grains are much higher than that of inferior grains. In modern rice cultivars, the numbers of grains per panicle has greatly increased, which is beneficial for yield improvement. However, the yield potential and grain quality are limited by poor grain filling of later flowered spikelets at lower branch of rice spikelets [5,6]. Therefore, studies on the reasons of poor grain filling of inferior spikelets are beneficial to improve rice yield for cultivars that have numerous spikelets on the panicle.

Starch, storage proteins and other constituents are the main accumulated reserve during rice grain development [7]. Major biological processes are believed to require a close coordination of gene expression among many important pathways in cereal grains [8]. During rice grain filling, sucrose produced in leaves is imported by the heterotrophic organs and used as carbon source in starch synthesis in the amyloplast [9,10]. In cytosol, sucrose is broken down into glucose and fructose. Then glucose/fructose are transformed into Glucose-6-P, Glucose-1-P, and finally into ADP-glucose. The ADP-glucose is used as the raw material in starch synthesis in amyloplast [11–13]. Sucrose synthase enzyme is the first key enzyme breaking sucrose into glucose and fructose [14]. Previous study found the sucrose synthase enzyme activity in superior grains was higher than that of inferior grains [15]. Zhu et al (2011) also reported that the gene expression of starch metabolism-related genes was higher in superior grains by using DNA microarray and real-time RT-PCR methods. Researches based on gene expression profile and protein 2-D electrophoresis profile also showed genes or proteins expressed differentially between the two kinds of grains [6,16–18].

RNA-Seq is a recently developed approach to study gene expression profiling that uses the next generation sequencing technologies, and provides a more precise measurement of gene transcripts dynamics on global scale in different tissues and biological contexts [19,20]. Recent studies also showed that RNA-Seq technology was highly reproducible for both technical and biological replicates, as compared with other methods like micro-array [21,22]. Studying transcriptome dynamics provided important insights into the functional elements of the genome, their expression patterns, and the regulation of transcribed regions in different tissues and under different conditions [23].

In this study, we investigated the dynamics of gene expression in four developing periods of rice superior and inferior grains by using RNA-Seq technique. In total, expressions of 19,442 genes were detected in one or more of the eight libraries constructed from superior and inferior grain samples. Genes involved in storage protein accumulation, sucrose and starch biosynthesis, plant hormone metabolism and cell cycle related genes were specifically examined and the potential mechanisms of poor filling of inferior grains were discussed.

## Materials and Methods

### Plant materials and sampling

*Oryza sativa spp*. *japonica* cv. Xinfeng 2 was planted in field (34°5’ N, 113°35’ E, 94m altitude) which belongs to Henan Agricultural University for field experiments purpose. No specific permissions were required for all field experiments in this study. The field studies did not involve endangered or protected species. Xinfeng 2 was a japonica rice cultivar developed by Guifeng Wang and cultivated in Huanghuai river basin, China. The superior grains was defined as spikelets positioned on the upper three primary branches, and the inferior grains was defined as spikelets positioned on the lower three proximal branches as described by Peng *et al* (2011). The flower day of spikelet was defined as 0 day after fertilization (DAF). Superior grains were sampled at 10, 15, 21, 27 DAF. After inferior spikelets flowered, which is normally 5~7 days later than superior grains [24], inferior grains were also sampled at 10, 15, 21, 27 days after inferior spikelet flowering. Both superior and inferior grains were separated from the panicle and frozen in liquid nitrogen for RNA extraction. Dynamic changes of grain weight of superior and inferior grains were measured at 5, 10, 15, 21, 27, 35DAF according to the method used by Peng *et al* [5].

RNA isolation, library construction, sequencing and digital tag profiling, quantitative real-time RT-PCR (Q-PCR) validation of RNA-Seq sequencing data were conducted by methods used by Peng et al (2013) [25]. Screening of differentially expressed genes was analyzed as described in Peng et al (2014) [26].

## Results

### Grain weight changes of superior and inferior grains

**Fig 1:**
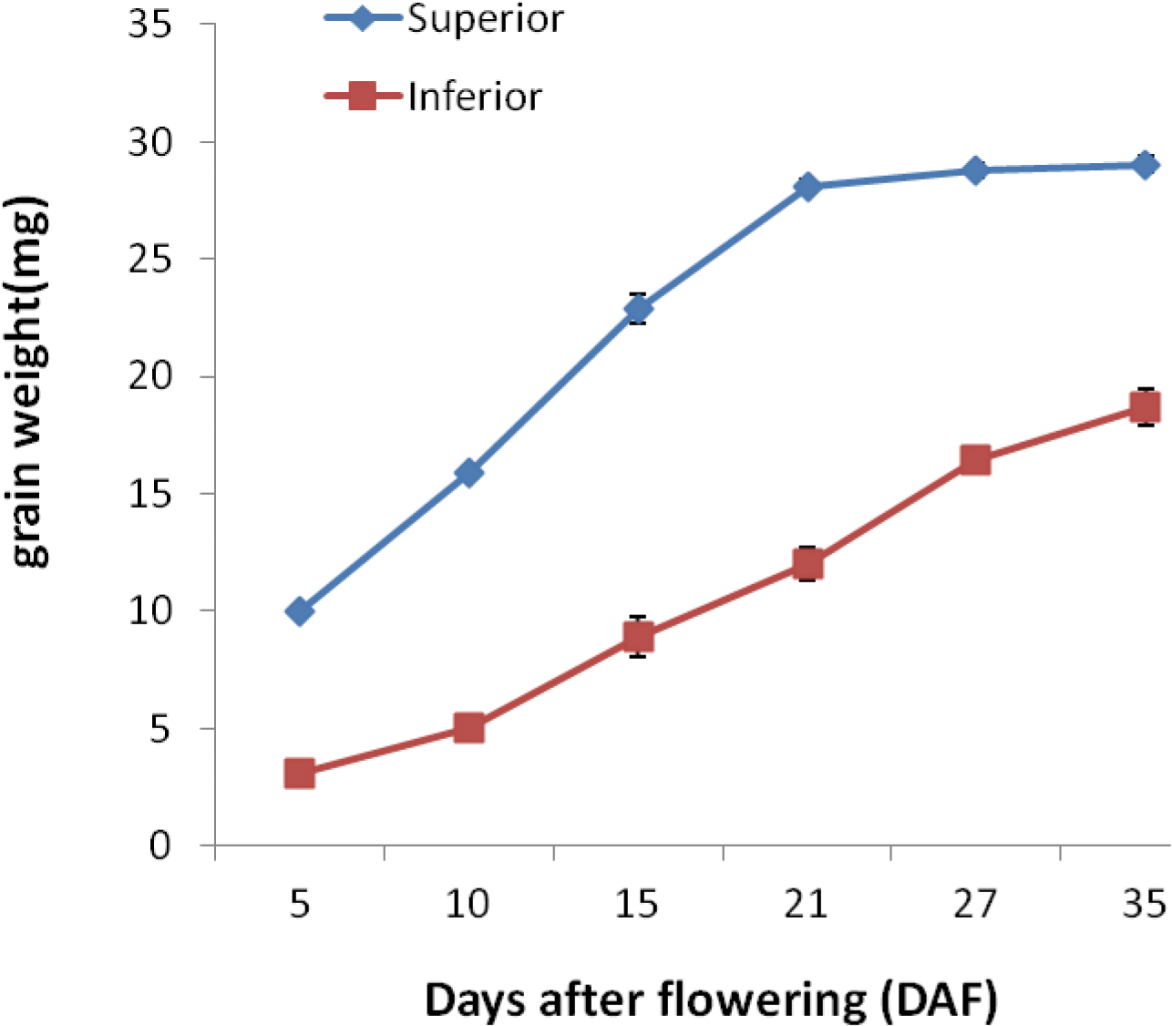
Grain weight of superior and inferior grains on the rice panicle. Grains were sampled at 5, 10, 15,21,27,35 days after fertilization, and the average dry weight of grains was measured.

During the grain filling process, superior grains showed rapid weight increase after fertilization. The grain weight reached stationary phase at 21DAF and did not change much till final stage. However, the inferior grains weight increased relatively slowly compared with that of superior grains and kept on increasing till late filling stage. Besides, the final grain weight was much lower than that of superior grains (See Fig 1).

### Summary of seed digital gene expression profiling

In the eight sample libraries, 3,181,415 to 6,025,486 raw tags were generated by high throughput sequencing. After removing low quality tags, 3,015,196 to 5,897,726 clean tags were obtained in each library. Of these clean tags, 82.07% to 91.96% could be mapped to rice genes (Rice Genome Annotation Project, Release 7), and 48.36% to 60.56% tags could be unambiguously mapped to known gene locus. Expressions of 10,799 to 16,657 genes were detected in each library, and 19,442 genes were detected in at least one library in total. The summary of the tags and gene number information for each sample was shown in Table 1. Expressions of each gene measured by transcripts per million (TPM) were listed in S1 Table. Six genes involved in starch synthesis, IAA synthesis and homeostasis and transcription related genes were chosen to validate the sequencing data by using Q-PCR method. The Q-PCR results showed that there were small differences between RNA-Seq data and Q-PCR, but the gene expression trends were similar between the two methods (See S1 Fig).

**Table 1:** Summary of sequencing and annotation results in the four filling stages of superior and inferior grains.

	10DAF_S	15DAF_S	21DAF_S	27DAF_S	10DAF_I	15DAF_I	21DAF_I	27DAF_I
Total tag number	4,961,639	6,025,486	3,559,090	3,181,415	5,862,147	3,536,663	3,492,932	3,397,235
Total clean tag number	4,801,042	5,897,726	3,377,893	3,015,196	5,644,440	3,289,946	3,246,054	3,194,649
Clean tag number mapped to gene	4,304,523	5,223,911	3,098,828	2,772,762	4,685,897	2,699,950	2,869,084	2,899,474
Percent of clean tag mapped to gene	89.66%	88.58%	91.74%	91.96%	83.02%	82.07%	88.39%	90.76%
Number of unambiguously mapped Tag	2,722,587	3,571,894	1,990,299	1,825,206	2,729,742	1,606,108	1,737,915	1,745,807
Percent of unambiguously mapped tag	56.71%	60.56%	58.92%	60.53%	48.36%	48.82%	53.54%	54.65%
Unknown clean tag number	261,076	445,393	155,811	157,501	446,758	334,124	188,274	167,664
Percent of unknown clean tag	5.44%	7.55%	4.61%	5.22%	7.92%	10.16%	5.80%	5.25%
Number of genes detected	14,967	13,681	12,148	10,799	16,657	15,201	14,484	13,111

Note: “DAF” indicates day after fertilization; “S” indicates superior grain; “I” indicates inferior grain.

### GO and KEGG analysis of expressed genes

**Fig 2:**
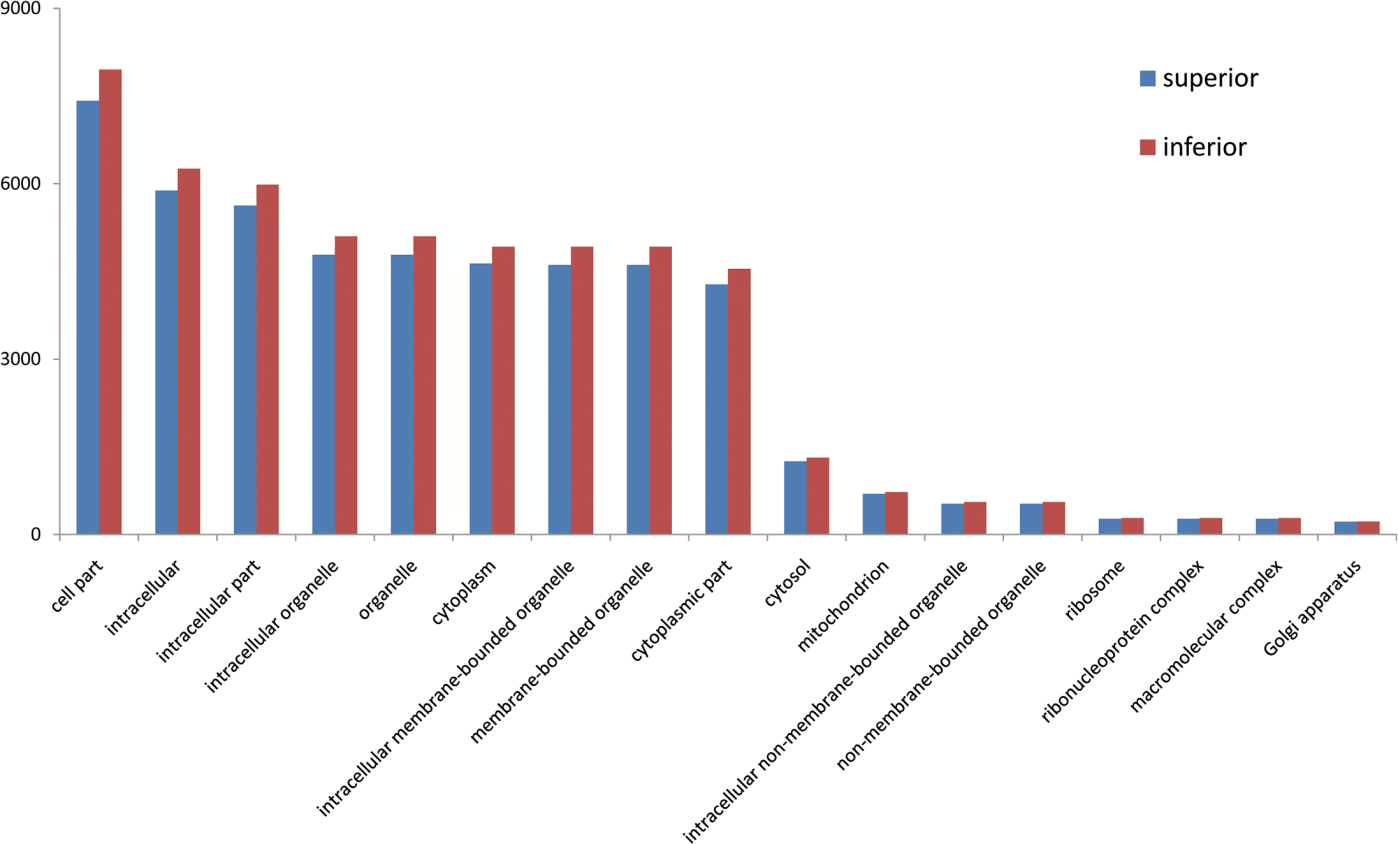
Gene Ontology (GO) enrichment analysis. The genes expressed in superior grains were taken as input list, and genes expressed in inferior grains were background list. All categories were classified according to their biological process, cellular component and molecular function. The blue bar indicates percentage of genes obtained in this study in each GO category, and the green bar indicates percentage of all annotated rice genes in each GO category.

In total, 16,622 and 18,521 genes were detected during development of superior and inferior grains, respectively. GO slim downloaded from Rice Genome Annotation Project (http://rice.plantbiology.msu.edu/) was used for the functional classification of these genes by cellular component, molecular function, and biological process categories. The inferior grains contained more genes in most GO terms of cellular component, molecular function, and biological process categories. Genes in superior and inferior grains were subjected to singular enrichment analysis by agriGO software [27] using genes in superior grains as input list and genes in inferior grains as background query list. The enrichment analysis showed that 18 GO terms were enriched in superior grains (Fig 2). These enriched GO terms all belonged to cellular component category, such as intracellular part, cytoplasm, intracellular, etc (S2 Table).

All the expressed genes in each library were compared between superior and inferior grains at the same period after flowering. As a result, 6,350, 6,704, 4,840 and 3,418 genes were defined as significantly differentially expressed by our screening standards at 10DAF, 15DAF, 21DAF and 27DAF, respectively. Pathway analysis were performed using the KEGG pathway database, and 3,789 genes were mapped on 115 known rice pathways including starch and sucrose metabolism (ko00500), fructose and mannose metabolism (ko00051), plant hormone signal transduction (ko04075), zeatin biosynthesis (ko00908), etc (S3 Table).

### Genes involved in starch and sucrose metabolism

Synthesis and accumulation of starch is the most important processes during rice grain filling, so we checked the transcription of genes involved in starch and sucrose metabolism in particular. Of the differentially expressed genes (DEGs) between superior and inferior grains, 146 genes were mapped on KEGG starch and sucrose metabolism pathway map (ko00500, see S3 Table).

**Fig 3:**
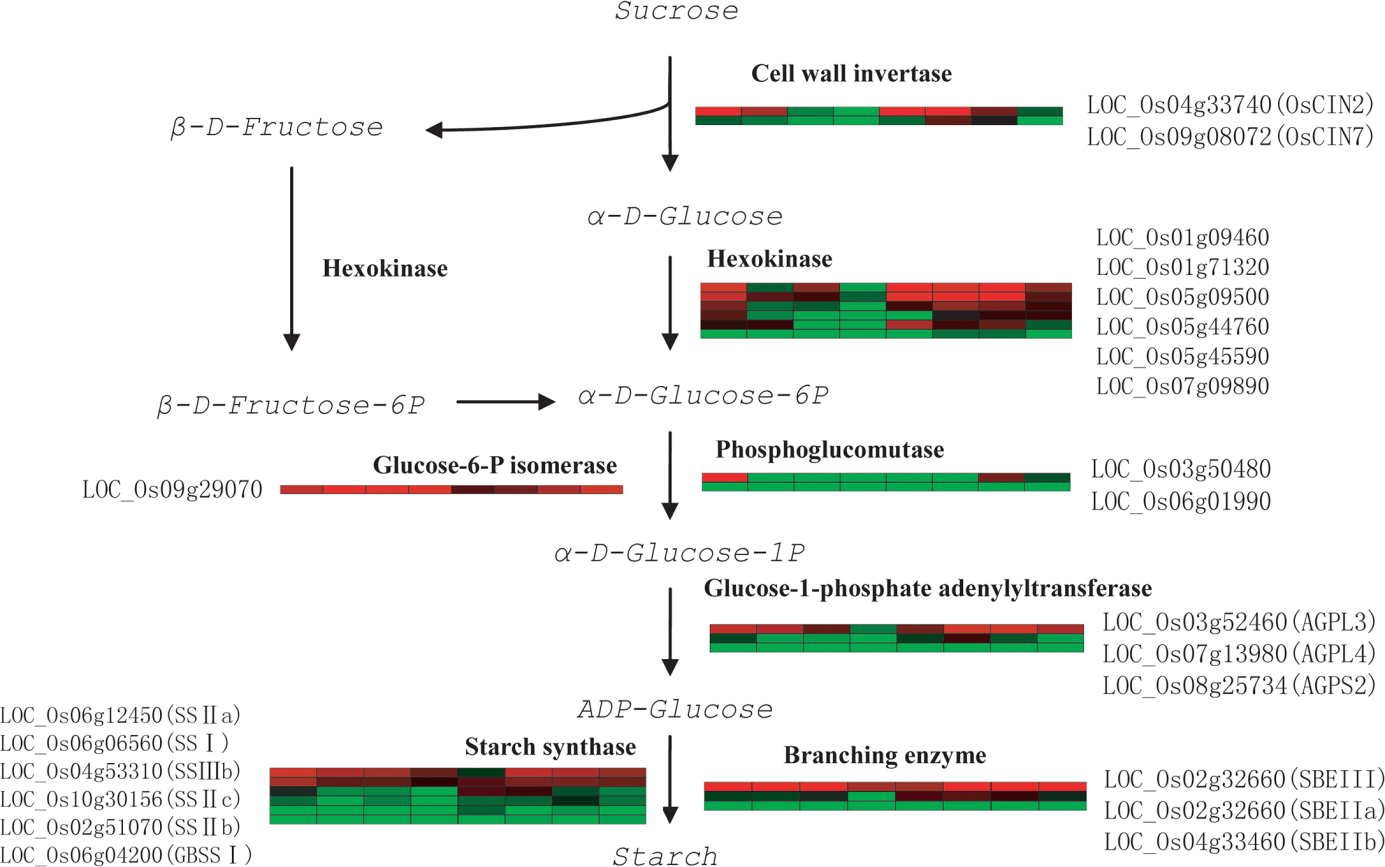
Schematic illustration of sucrose to starch synthesis pathway. Flow of substrates and products is indicated by arrows and enzymes catalyze the reaction are labeled beside arrows. Heat maps of the genes encoding the enzymes are placed beside the enzymes. Each grid indicates a sampled period. The first four grids indicate superior grains of 10DAF, 15DAF, 21DAF, 27DAF, and the last four grids indicate inferior grains of 10DAF, 15DAF, 21DAF, 27DAF. Expression of genes (in TPM) was subjected to log2 conversion. With those zero expression genes, zero was converted with 0.01(in TPM).

In rice grains, hydrolysis of sucrose into glucose and fructose is catalyzed by cell wall invertase [1,28]. Eight members of cell wall invertase were found in the rice genome till now, but only OsCIN1, OsCIN2, OsCIN4 and OsCIN7 were expressed in developing seeds [29]. In our RNA-Seq data, only OsCIN2 (LOC_Os04g33740) and OsCIN7 (LOC_Os09g08072) were detected. Of the two cell wall invertase genes, OsCIN2 was the mainly expressed one, which was also designated as GIF1 gene [1]. In superior grains, the expression of OsCIN2 gene was higher at 10DAF, and dropped afterwards till no signal at 27DAF. While, in inferior grains, OsCIN2 gene was lower at 10DAF, but reached peak expression at 15DAF and dropped afterwards. The expression of OsCIN2 gene in inferior grains was all higher than superior grains at 15, 21, 27DAF (See Fig 3).

Glucose is transformed into α-D-Glucose-6P by hexokinase, and β-D-Fructose is turned intoβ-D-Fructose-6P by hexokinase and then to α-D-Glucose-6P by Glucose-6-P isomerase. We checked the expression of these two enzymes and found several hexokinase members were expressed in developing grains, and the inferior grains showed higher expression in all the four periods we sampled. As to the Glucose-6-P isomerase gene, expression was comparable at the same level at 10DAF, and slightly higher in inferior grains afterwards.

α-D-Glucose-6-P is transformed into α-D-Glucose-1-P by phosphoglucomutase[30]. In our RNA-Seq data, expressions of two phosphoglucomutase gene members were detected. Of them, LOC_Os03g50480 was the mainly expressed. In superior grains, phosphoglucomutase (LOC_Os03g50480) was expressed at higher level at 10DAF, but dropped afterwards till zero expression at 27DAF. While, in inferior grains, phosphoglucomutase (LOC_Os03g50480) was expressed at lower level at 10DAF and 15DAF. But at 21DAF and 27DAF, the expression became higher (See Fig 3).

The next step in starch synthesis is α-D-Glucose-1-P catalyzed by glucose-1-phosphate adenylyltransferase and turned into ADP-glucose [31]. In RNA-Seq data, expression of three glucose-1-phosphate adenylyltransferase subunit genes (AGPL3, AGPL4 and AGPLS2) was analyzed. The expression pattern of the three genes all showed higher expression in superior grains at early filling stage and higher in inferior grains at late filling stage (see Fig 3).

Starch in the rice endosperm generally consists of amylose and amylopectin. Biosynthesis of starch is catalyzed by starch synthase and 1,4-alpha-glucan-branching enzyme, which are in charge of amylose and amylopectin synthesis, respectively. In our data, expression of six starch synthase genes were detected by RNA-Seq, but only soluble starch SSIIa (LOC_Os06g12450) and SSI (LOC_Os06g06560) were highly expressed (>100TPM). At 10DAF in superior grains, expression of SSIIa (LOC_Os06g12450) and SSI (LOC_Os06g06560) were 602.16 and 282.02 TPM, respectively. The expression of these two genes dropped down along with the process of grain filling, and reached the lowest at late filling stage. While, in inferior grains, these two genes were 21.97 and 75.65 TPM at 10DAF, respectively. Although expression of other starch synthases was detected, and these starch synthases expressed higher in the inferior grains, but due to their lower amount compared to that of starch SSIIa (LOC_Os06g12450) and SSI (LOC_Os06g06560), the expression of starch synthases in superior grains was much higher in early filling stage. At late filling stage, expression of starch synthases was higher in inferior grains (see Fig 3).

As to the amylopectin synthesis, branching enzyme (BE) catalyzes the formation of branch points by cleaving the a-1,4 linkage in polyglucans and reattaching the chain via ana-1,6-glucan linkage[11]. Expression of three 1,4-alpha-glucan-branching enzyme genes were found during grain filling, and SBEIII (LOC_Os02g32660) was expressed far more higher than the other two members, SBEIIa and SBEIIb (LOC_Os04g33460 and LOC_Os06g26234). The SBEIII (LOC_Os02g32660) expression level was much higher in superior grains at 10DAF and 15DAF than inferior grains. But at late filling stage, its expression was higher in inferior grains and reached comparative level as that of superior grains (see Fig 3).

Starch is synthesized in amyloplast, and several translocater gene plays important roles during the starch accumulation process [13]. The *BT1* gene was suggested to transport ADP-glucose from cytosol to plastid [32]. Of the three *BT1* genes in rice, *BT1-2* (LOC_Os02g10800) was the mainly expressed in grains (data from RiceXPro, http://ricexpro.dna.affrc.go.jp/) [33]. In superior grains, *BT1-2* was expressed at 84.36TPM at 10DAF and dropped afterward to 16.58TPM at 27DAF, while, in inferior grains, its expression was 7.8TPM at 10DAF and reached to 129.49TPM at 15DAF and then dropped down to 11.27TPM at 27DAF. Another transporter, *GPT*3 (Glucose-6-P translocator) gene (LOC_Os05g07670) was also detected in the sequencing data, which transports Glucose-6-P through the plastid membrane [13]. The expression pattern showed that inferior grains expressed higher level of *GPT*3 in all the four periods we sampled.

### Expression pattern of genes controlling grain development

**Fig 4:**
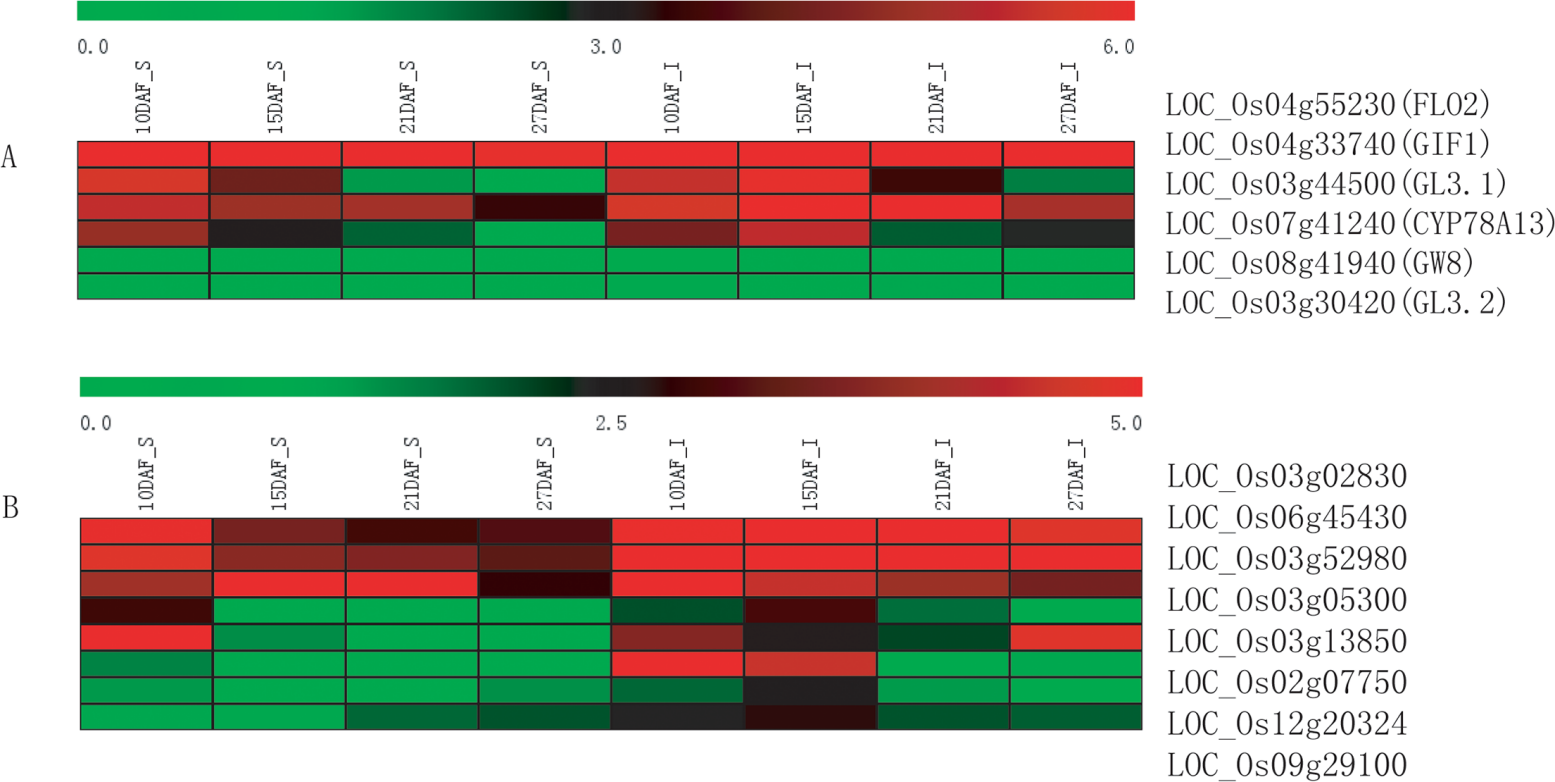
Heat map representation of grain size controlling and cell cycle related genes. “A” indicates grain size controlling genes expression. “B” indicates cell cycle related genes expression. “DAF” indicates day after flowering. “S” indicates superior grains, and “I” indicates inferior grains. Expression of genes (in TPM) was subjected to log2 conversion. With those zero expression genes, zero was converted with 0.01(in TPM).

Till now, a number of genes controlling rice grain development had been cloned. Of those cloned genes, *GIF1*, *GW5*, *GW8*, *GS3*, *GS5*, *FLO2*, *OsTGW6*, *CYP78A13*, *GL3*.*1*were found to be involved in grain size development [1,34–41]. In our RNA-Seq data, six of those genes were present in the samples (See Fig 4A). GIF1 (LOC_Os04g33740) was a gene controlling rice grain filling, and overexpression of GIF1 increase grain production [1]. GIF1 was also designated as cell wall invertase or OsCIN2 (See Fig 4A). *GW8* (LOC_Os02g14720) was synonymous with *OsSPL16*, and loss-of-function mutation in *GW8* resulted in slender grain. In superior grains, expression of *GW8* was not detected. While in inferior grains, it was expressed at low level at 10DAF and 15DAF. FLO2 played important role in controlling rice grain size and quality, and loss-of-function in *FLO2* caused down-regulation of genes involved in starch and storage production in endosperm[35]. *FLO2* (LOC_Os04g55230) was expressed at higher level in superior grains at 10DAF and dropped afterwards, whereas, inferior grains showed lower expression at 10DAF and relatively higher expression at 15DAF and 27DAF compared with superior grains. *CYP78A13* was a cytochrome P450 gene which could promote cell proliferation, and over-expression of *CYP78A13* or its paralogue (*GL3*.*2*) could increase grain size [37]. Expression of these two genes was checked in our RNA-Seq data and we found *CYP78A13* (LOC_Os07g41240) in superior grains was expressed higher than inferior grains at 10DAF, but lower at afterward periods. While, *GL3*.*2* (LOC_Os03g30420) was expressed at very low level in inferior grains, but undetected in superior grains. *GL3*.*1* was another gene controlling rice grain length [38]. The mutant allele had weaker dephosphorylation activity than wild type, thus accelerated cell division and resulted in longer grains. Expression of *GL3*.*1* was higher in inferior grains in all the four periods we sampled, which indicated a stronger dephosphorylation activity in inferior grains and would result in shorter grains.

To investigate the genes involving grain size development, we further checked the expression of eight genes involved in cell cycle or cyclin related proteins. The expression of these cell cycle related genes were higher in inferior grains, especially at middle and late period of grain filling (see Fig 4B). The results indicated that at 10DAF, inferior grains were still in active cell division stage and superior grains had completed cell division process and mainly involved in starch synthesis.

### Dynamic expression of prolamins, glutelins and albumins storage protein genes

**Fig 5:**
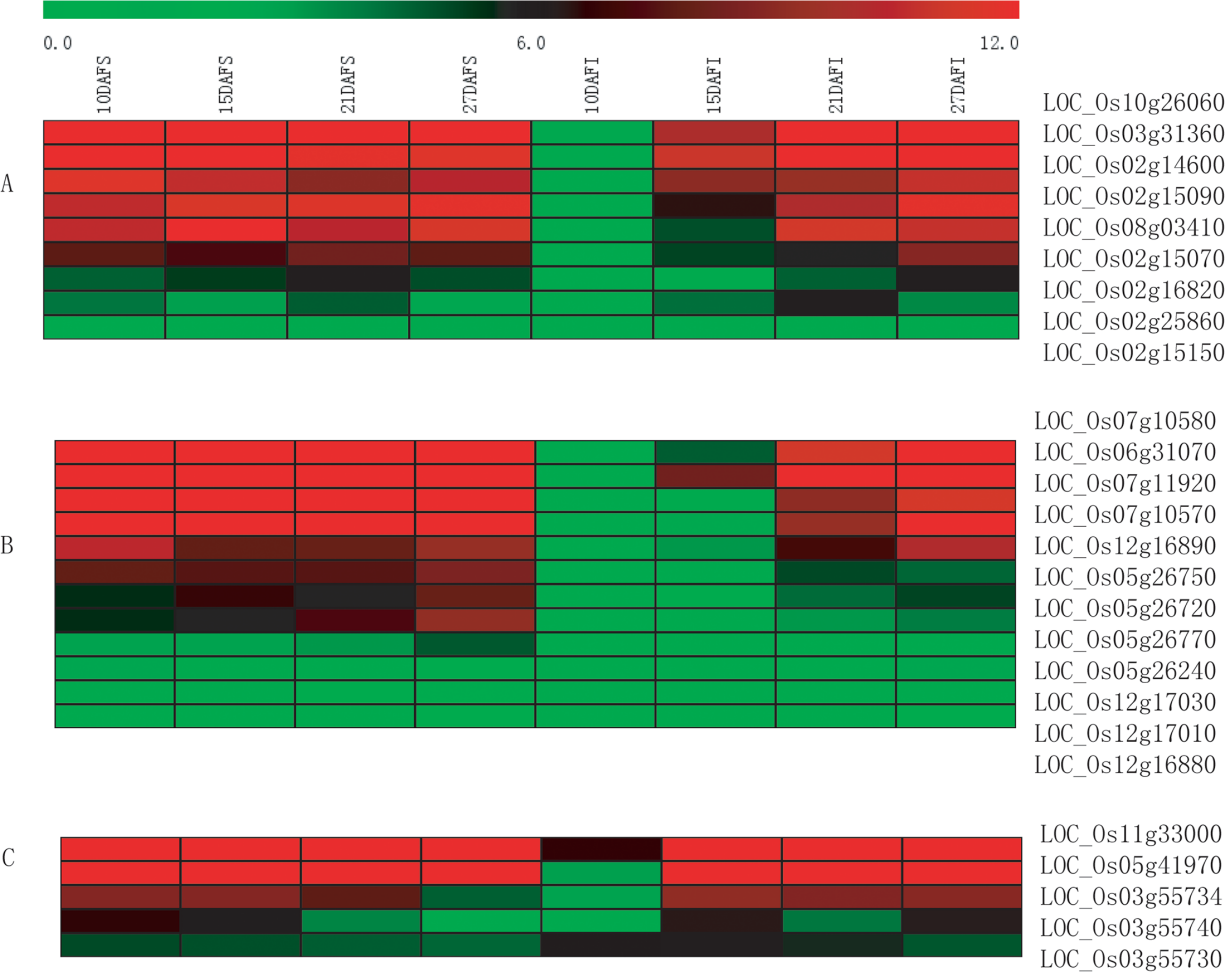
Heat map representation of seed storage protein genes expression. “A” indicates glutelin genes, “B” indicates prolamin genes, and “C” indicates albumin genes. “DAF” indicates day after flowering. “S” indicates superior grains, and “I” indicates inferior grains. Expression of genes (in TPM) was subjected to log2 conversion. With those zero expression genes, zero was converted with 0.01(in TPM).

Seed storage proteins are the main source of nitrogen accumulation during seed maturation, which include glutelins, prolamins, albumins, and globulins. In rice, glutelin is the major storage protein, accounting for 60–80% of total seed protein. While, prolamin makes up about 20–30% of total seed protein [42]. In total, nine glutelins genes and twelve prolamin genes were detected in our RNA-Seq data. Of the nine glutelin genes, LOC_Os10g26060 was the most highly expressed one, which reached peak value of 61854.53TPM at 21DAF in superior grains. However, in inferior grains, expression of LOC_Os10g26060 was negligible (<10TPM) at 10DAF and 15DAF, and gradually increased and reached peak value (42758.06TPM) at 27DAF (see Fig 5A). In superior grains, the prolamin genes were highly expressed during the filling stage, but in inferior grains, the expression level of prolamin genes was extremely low at early filling stage and increased until late filling stage. Moreover, even at late filling stage, the highest expression level was relatively low compared with the peak value of superior grains (see Fig 5B). The same trend was observed in albumin genes expression. The only difference was that at 27DAF, expression level of albumin genes in inferior grains reached peak value which was higher than that of superior grains (see Fig 5C).

## Discussion

Rice grain filling is a highly complicated process, and the accumulation of starch and storage proteins is regulated starting at the mRNA level [8]. The RNA-Seq technique provided a powerful tool on transcriptome level for studying and understanding genes involved in grain development and grain filling. Starch accounts for 80–90% of the final weight of rice grains, thus starch synthesis plays a central role during grain filling [16]. In total, expression of 147 genes involved in starch synthesis was detected in our RNA-Seq data. It was reported that sucrose synthase was the key limiting factor affecting poor filling of inferior grains [15]. But in our data, the expression level of sucrose synthase (LOC_Os02g58480) was higher in inferior grains in all the four grain filling periods we sampled. One possible reason might be the timing of sampling. Ishimaru et al. (2005) reported that in rice grains, the expression of sucrose synthase reached peak at about five days after fertilization, and then dropped afterward. While in inferior grains, the peak appeared at nine or fifteen days after fertilization according different gene members. As our sampling time started from ten days after fertilization, so the relative expression level was higher in inferior grain because it might be dropped already in superior grains at that time.

Sucrose is the most important form in carbohydrate transportation from source to sink organ [9]. Eight enzymes catalyze the key reactions in the process of sucrose to starch transformation (Fig 3). At ten days after fertilization, seven of the eight key enzyme genes in inferior grains were expressed at comparable level as those of superior grains, except for phosphoglucomutase. Phosphoglucomutase catalyzes the transformation of α-D-Glucose-6P to α-D-Glucose-1P, which is a key step in providing the raw material ADP-Glucose used in starch synthesis. Glucose-1-phosphate adenylyltransferase and starch synthase are the last two key enzymes in sucrose to starch transformation. It has been reported that the low activities of starch synthesis-related enzymes might be the key limiting factor leading to the poor filling of inferior grains [15,16]. But in this study, the expression of genes in inferior grains involved in sucrose to starch synthesis pathway was not much lower than superior grains at early filling stage except phosphoglucomutase coding gene. Expression of phosphoglucomutase gene reached peak at 27 days after fertilization in inferior grains, and before this stage the gene expression was relatively low compared with superior grains. Therefore, low expression of phosphoglucomutase gene might be a key limiting factor affecting inferior grain filling at early filling stage.

On the other hand, the inferior spikelets flowered five to seven days later than superior spikelets [5]. This would cause a lag shift of grain filling process in inferior spikelets, and it was verified by shift of storage proteins accumulation and grain filling rate peak. Genes involved in cell cycle or cyclin related genes were all found higher in inferior grains in each of the filling stages we sampled, which indicated that the inferior grains were still in active process of cell division. This result was supported by previous report that the cell division rate was higher in superior grains at early filling stage, and the inferior grain cell division rate reached peak 16–20 days after fertilization according to different varieties [3]. As to the genes that controlling grains size, *CYP78A13* and *GL3*.*1* were two genes which could promote or repress cell proliferation. At early filling stage, expression patterns of the two genes were both disadvantageous to cell proliferation in inferior grains. So, at early filling stage, sink organ capacity might be another limiting factor of inferior grain filling. At late filling stage, when the cell number and gene expression reached relatively higher level in inferior grains, the photosynthetic rate of leaves and sucrose content in grains dropped sharply [43,44]. At this filling stage, the superior grain filling almost finished, but in inferior grains, due to the reduction of raw material in starch synthesis, accumulation of starch was inevitably affected.

## Supporting Information

S1 Fig Q-PCR validation of superior and inferior grains sequencing data. Gene names (Locus No.) were labeled on top. The left ones were data from Q-PCR, and the right ones were data from RNA-seq sequencing.
(PDF)

S1 Table Lists of all genes expression detected in RNA libraries (in TPM). (XLS)

S2 Table GO enrichment analysis of genes differentially expressed between rice superior and inferior grains. (XLS)

S3 Table KEGG pathway classification of expressed genes in superior and inferior grains. (XLS)
