# Supplementary material for: Dynamic Analysis of Gene Expression in Rice Superior and Inferior Grains by RNA-Seq
Source: PLoS One. 2015 Sep 10;10(9):e0137168. doi: 10.1371/journal.pone.0137168 (PMC4565701; doi:10.1371/journal.pone.0137168)
Supplement: S1 Fig — Gene names (Locus No.) were labeled on top. The left ones were data from Q-PCR, and the right ones were data from RNA-seq sequencing. (PDF) [file pone.0137168.s001.pdf]

LOC_Os06g12450（SSIIa）

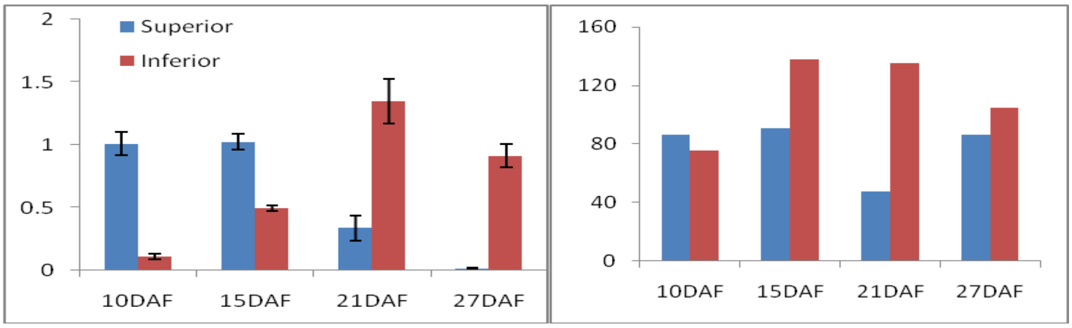

LOC_Os06g46270（NAC21/22）

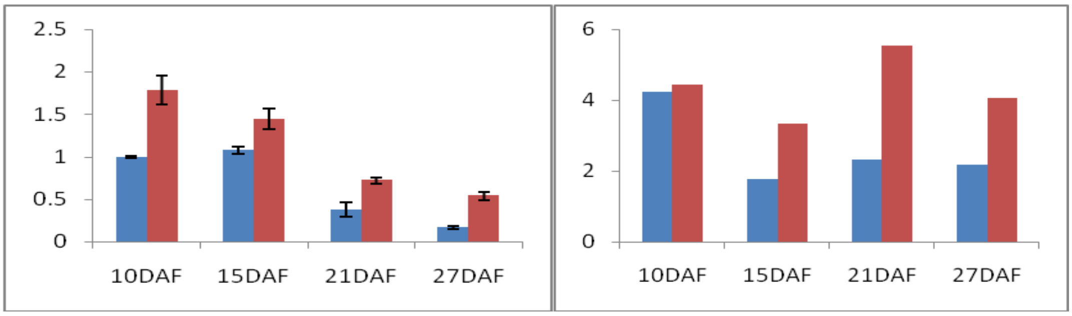

LOC_Os02g06910（ARF6）

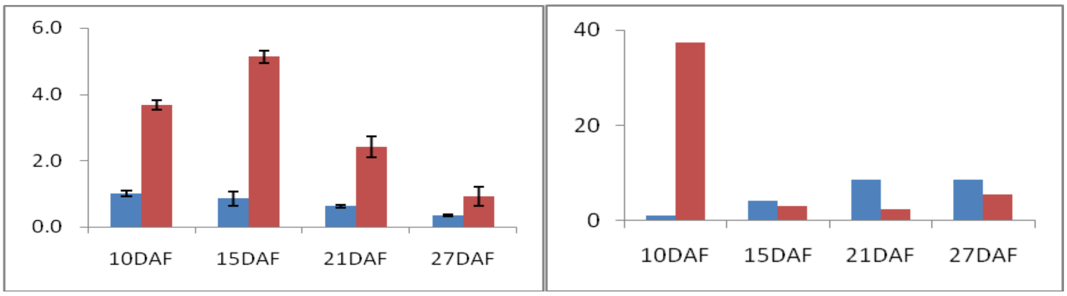

LOC_Os12g08780（YUC11）

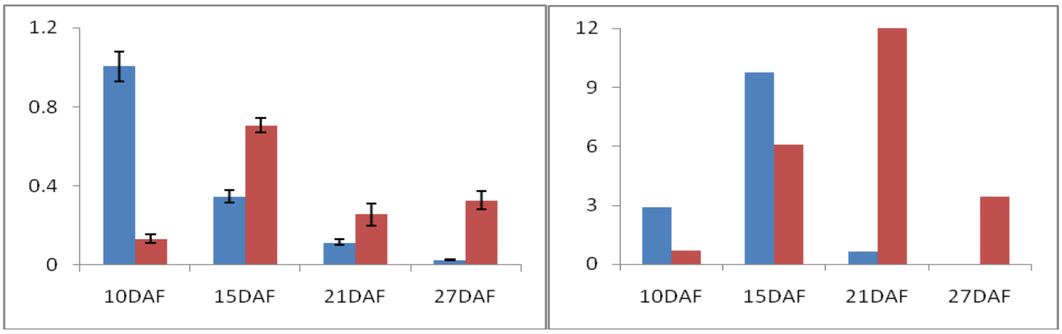

LOC_Os05g07720（TAR1）

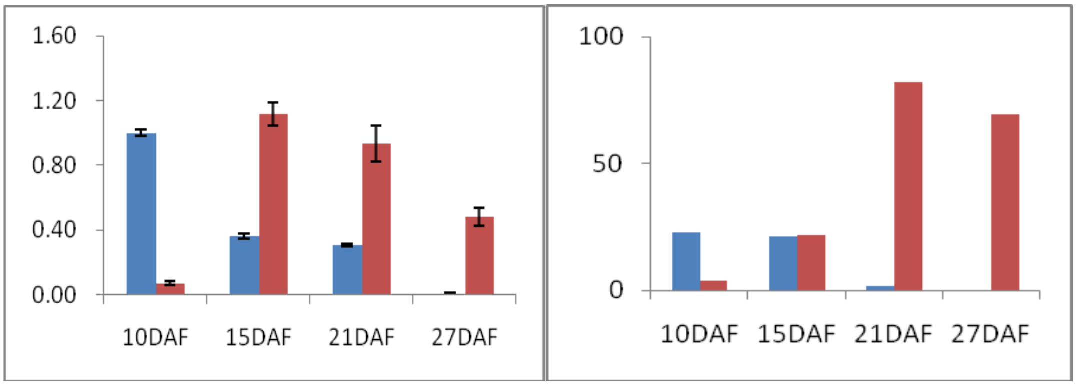

Figure S1 Q-PCR validation of superior and inferior grains sequencing data. Gene names (Locus No.) were labeled on top. The left ones were data from Q-PCR, and the right ones were data from RNA-seq sequencing.
